# Implication of nutrition in severity of symptoms and treatments in quality of life in Parkinson’s disease: a systematic review

**DOI:** 10.3389/fnut.2024.1434290

**Published:** 2024-10-08

**Authors:** Naia Ayo Mentxakatorre, Beatriz Tijero, María Ángeles Acera, Tamara Fernández-Valle, Marta Ruiz-Lopez, Juan Carlos Gómez-Esteban, Rocio Del Pino

**Affiliations:** ^1^Neurodegenerative Diseases Group, Biobizkaia Health Research Institute, Barakaldo, Spain; ^2^Department of Surgery, Cruces University Hospital, Barakaldo, Spain; ^3^Department of Neurology, Cruces University Hospital, Barakaldo, Spain; ^4^Department of Neuroscience, University of the Basque Country (UPV/EHU), Leioa, Spain

**Keywords:** deep brain stimulation, levodopa/carbidopa intestinal gel, nutritional alterations, nutritional status, Parkinson’s disease, quality of life, severity of symptoms

## Abstract

Parkinson’s disease (PD) is characterized by motor and non-motor symptoms. Nutritional alterations are one of the non-motor symptoms that most influence the quality of life (QoL) in PD.

**Objective:**

Therefore, this review aims to evaluate whether nutritional alterations are related either to the severity of motor and non-motor symptoms through the gut-brain axis or to the different treatments for PD and whether all of this, in turn, impacts the QoL of patients.

**Methods:**

A systematic review was carried out in MEDLINE and EMBASE databases, and Mendeley from 2000 to June 2024, searching for articles related to nutritional alterations in PD that alter patients’ QoL. A total of 14 articles (2,187 participants) of 924 records were included.

**Results:**

Among the 14 studies examined, two investigated the relationship between nutritional status and QoL in patients with PD. Poor nutritional status was associated with lower QoL scores. Four studies explored the connection between nutritional status and its impact on both motor and non-motor symptoms (psychiatric disturbances, cognitive impairment, and fatigue), revealing a link between nutritional status, activities of daily living, and the severity of motor symptoms. Three studies identified changes in body weight associated with the severity of symptoms related to mobility issues in PD patients. Three studies investigated the relationship between different PD treatments and their interaction with changes in weight and energy metabolism, highlighting that weight loss in the early stages of PD needs adequate monitoring of different treatments, as well as the interaction between the central and peripheral nervous systems in regulating these processes. Finally, two studies investigated how gastrointestinal alterations and changes in the microbiota were related to cognitive status, thus identifying them as risk factors and early signs of PD.

**Discussion:**

The systematic review highlighted the significant relationship between nutritional status and QoL in patients with PD, as well as how the PD treatments influenced their weight. An association was also observed in the gut-brain axis, where adequate nutritional status influenced the balance of intestinal microbiota, slowing cognitive decline, improving activities of daily living, and the QoL of PD patients. It is confirmed that the nutritional status of patients influenced both motor and non-motor symptoms of the disease, and therefore their QoL.

## 1 Introduction

Parkinson’s disease (PD) is the second most common neurodegenerative disease after Alzheimer’s disease (Table 1). The incidence is 1–4% of the population between 60 and 80 years, respectively (1). In addition to the loss of neurons in the substantia nigra, which causes a lack of dopamine in the body, PD is characterized by motor symptoms such as bradykinesia, tremor at rest, rigidity, and postural instability, as well as the appearance of accumulations of the protein alpha-synuclein in the central nervous system and other neural structures. The non-motor symptoms are more difficult to identify and treat since involve several anatomical systems in different regions of the nervous system (noradrenergic and serotonergic neurons of the brainstem, dopaminergic in the mesolimbic and mesocortical circuits). The loss of smell, gastrointestinal symptoms (constipation, loss of appetite, drooling, dysphagia, gastroesophageal reflux), and cardiovascular and urogenital symptoms are common non-motor symptoms in PD (2) significantly influence the patient’s quality of life (QoL) (3). All these symptoms could be described as prodromal or initial signs of the disease, and many of them may appear years before its definitive diagnosis.

PD patients are very vulnerable from the nutritional point of view since they usually lose weight due to the imbalance between the intake made by the patient and their energy consumption continued over time. In addition, other factors such as symptoms related to dysautonomia (sialorrhea, dysphagia, and constipation) may intervene in this vulnerability. There is also a high incidence of mood disorders (depression, anxiety, abulia, and apathy) (4) and up to 85% of those affected have hyposmia or anosmia from the early stages of the disease, which affects appetite (5). Therefore, the nutritional status of patients is important since there is a decrease in food intake and appetite from the early stages of PD.

Weight loss has therapeutic and poor prognostic implications. According to Jimenez et al. (3) the lower the weight, the higher the incidence of motor complications of dopaminergic treatment (dyskinesias) and the risk of general deterioration with complications. Dysphagia is also a serious condition in PD patients, presenting an increased risk of malnutrition, dehydration, pneumonia, and even death (6). Sepúlveda-Contardo et al. (7) described the aspects of QoL that affected to PD patients with dysphagia or dysarthria. They found a decrease in their QoL related to their diet and the capacity of speaking, reporting fatigue while eating or speaking, embarrassment in social activities, and extended feeding periods, among others (7).

Another aspect to take into account in the nutritional status of PD patients is the intestinal microbiota-brain axis in the pathogenesis and severity of PD. The prolonged alteration of the intestinal microbiota could produce an alteration in the intestinal barrier, and local and systemic inflammation, impacting the blood–brain barrier and causing neurodegeneration (8). Therefore, this review aims to evaluate whether nutritional alterations are related either to the severity of motor and non-motor symptoms through the gut-brain axis or to the different treatments for PD and whether all of this, in turn, impacts the QoL of patients.

## 2 Methods and materials

This review followed the Preferred Reporting Items for Systematic Reviews and Meta-Analyses checklist (PRISMA) (Supplementary material S1). The abbreviations are specified in Supplementary material S2.

To prepare this systematic review we have used the PICOS framework: patients with PD in which we will analyze if there is a relationship between their nutritional status, severity of symptoms, the different treatments received and their impact on their quality of life. Could we carry out an intervention on nutritional status that would improve the severity of the symptoms and this in turn would impact their quality of visa, improving it?

### 2.1 Study selection and procedure

The study included all empirical studies that met the following inclusion criteria: (1) Studies published from 2000 to June 2024; (2) Studies published in a peer-reviewed English or Spanish language journal; (3) Studies including human subjects older than 18 years. The exclusion criteria were as follows: (1) Duplicated studies; (2) Systematic reviews and/or narrative reviews; (3) Clinical trials; (4) Case reports; (5) Conference papers and abstracts; (6) Studies with animals; (7) Studies with participants with other diseases different to PD; (8) Studies including variants of PD; (9) Studies not focused specifically on the QoL and nutritional alterations.

**Figure 1:**
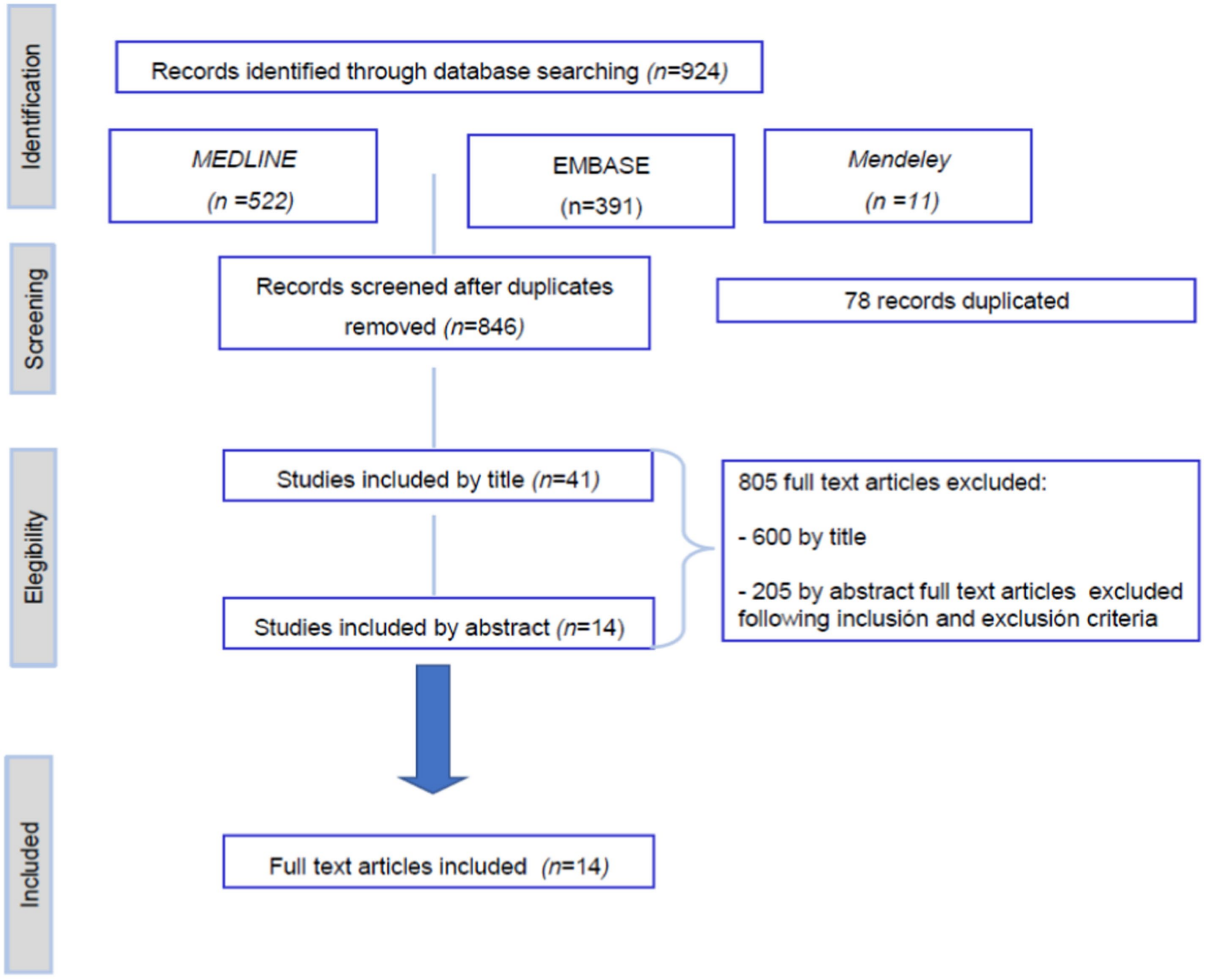
Flow diagram showing the process of study selection.

The search was performed in MEDLINE and EMBASE databases, and Mendeley, and the search was finished in June 2024. The keywords used for each search were: *Parkinson’s disease, quality of life, nutritional alterations, nutritional status, nutrition disorders, nutritive value, Deep brain stimulation, and dysphagia*. The specific keyword and mesh strategy are explained in the Supplementary material S3. Two experienced reviewers screened separately the search results using the inclusion and exclusion criteria explained above, following the subsequent steps: title and abstract screening, followed by full-text screening. When the judgments of any of the reviewers were not similar, the discrepancies were explained, and a common decision was taken. The bibliographic databases yielded 924 references in total (Figure 1).

### 2.2 Data extraction and outcome

Two experienced reviewers used a preformatted Excel sheet to extract data for the prespecified relevant data and outcomes for each included article, (1) Total sample size; (2) Percentages of males; (3) Sample size included in the PD and control group; (4) Age of each group; (5) Objective of the study; (6) Methods/Measures; (7) Results (Table 1).

**Table 1:** Outcomes and results of selected studies.

Study	Variables				Analysis	Results
	PD clinical status	Nutritional status	Cognition	Others		
Nutritional status and Qol						
Fernandez et al. (9)	–	Weight, height, BMI, AC, TCS	–	PDQ-39	Pearson’s correlation, Multiple linear regression	The perception of QoL showed lower scores for body discomfort (75.3+/−16.6), social support (62.7+/−15.7), and mobility (61.0+/−23.6). Significant correlation between the QoL and age (*p* = 0.048), and BMI (*p* = 0.047), suggesting no influence of gender and nutritional status.
Sheard et al. (10)	UPDRS, H&Y, Mobility, stigma, social support	PG-SGA, MNA, BMI	ACE-R, EWB,	PDQ-39, ADL, LEDD	Mann–Whitney U test, Kruskal Wallis test, Friedman’s Two-Way ANOVA, and Spearman’s correlations.	Phase I QoL was poorer in the malnourished, particularly for mobility and ADL domains. There was a significant correlation between PG-SGA and PDQ-39 score (Phase I *p* = 0.000; Phase II *p* = 0.002). In phase II, no significant difference in PDQ-39 total or sub-scores was observed between the INT and SC groups; however, there was significant improvement in the emotional well-being domain for the entire group (*p* = 0.12).
Nutritional status and motor and non-motor symptoms						
Nagano et al. (11)	H&Y	CONUT (Alb, TLC, T-cho), BMI, dysphagia	–	Days and route hospitalization, weight on admission and discharge, ADL (FIM), PD drugs.	Mixed-effect model (linear and piecewise linear model)	A poor nutritional status was significantly associated with a poor ADL in PD.
Chia-yi Lien et al. (12)	UPDRS H&Y	MNA	MMSE	Biochemistry (vit D, B12, and folate levels, hs-CPR)	*χ*^2^ test, *t*-test linear regression	PD patients at risk of malnutrition status had higher UPDRS (*p* = 0.046), and impaired cognitive state, poorer memory (*p* = 0.002), and calculation (*p* = 0.010). PD patients with abnormal vitamin D levels had a significantly higher hs-CPR level (*p* = 0.046) which influenced on cognitive function of PD.
Fereshtehnejad et al. (13)	UPDRS, H&Y	MNA, MAC, CC	HADS	FSS, PDQ-39, ADL	The Kolmogorov–Smirnov, Spearman correlation. In the subgroup analysis, a *t*-test, Mann–Whitney U test, and multivariable analysis using a stepwise linear regression model	A higher score on the UPDRS scale and a longer duration of PD were associated with lower scores on the MNA scale. The median score of the H&Y stage was significantly higher in the PD with abnormal nutritional status (*p* < 0.001). More severe anxiety (*p* < 0.002), depression (*p* < 0.001), and fatigue (*p* < 0.001) were observed in PD patients with abnormal nutritional status. Except for stigma, all the other domains of the PDQ-39 were significantly correlated with the total score of MNA.
Sheard et al. (14)	UPDRS, H&Y, SCOPA-AUT, FOG-Q	SGA, PG-SGA	ACE-R, BDI, STAI, MMSE	MCAS, LEDD	Mann–Whitney U test, *χ*^2^ test, univariable and multivariable logistic regression.	More of the malnourished were elderly and had more severe diseases. UPDRSII, III scores and LEDD, body weight were significantly higher in the malnourished. Older age at diagnosis, higher LEDD and body weight, greater UPRDSII score lower STAI score, and higher BDI score as significant predictors of malnutrition. Living alone and higher BDI and UPDRSIII scores were significant predictors of higher log-adjusted PG-SGA scores.
Nutritional status and PD severity.						
Femat-Roldán et al. (1)	UPDRS, H&Y	BMI, LM, FFM, MSM, BFM, %BF, ICW, ECW, BMR, WC, VFA, BMC, BCM, AC, RMR	–	LEDD	*χ*^2^ test, *t*-test	The PD group had lower body mass, a lower percentage of whole-body mass, and a better rate of metabolic preposition compared with controls, with no significant differences in musculoskeletal mass. PD patients with postural instability and instability when walking had lower body fat parameters, increasing free fat mass, and higher RMR.
Pisciotta et al. (15)	UPDRS (ON)	Weight, height, BMI, Total and regional adiposity, total fat (android and gynoid), MNA	MMSE	LEDD ADL GDS blood sample ESR	ANOVA, *χ*^2^ test, linear model regression	Nutritional status drives the association between total and regional adiposity and disease severity in PD. More motor severity less total body fat in kg and %, % android fat, trunk-limb fat ratio, trunk-leg fat ratio, and android-gynoid fat ratio.
Bril et al. (16)	MDS-UPDRS (ON) H&Y, ROMEIII, Sniffin Sticks	BMI, MNA, being eating.	MoCA, BDI, QUIP	LEDD, PDSS-2, Yale physical activity survey	Pearson’s correlation, *χ*^2^ test, squared error of approximation RMSEA, comparative fit index CFI, Index of NFI normalized fit.	Longer disease duration was negatively related to nutritional status (*p* = 0.01). UPDRS II, III score was associated with reduced cognitive function (*p* = 0.01), which was positively related to nutritional status (*p* = 0.01). Nutritional status was positively related to body weight (*p* < 0.01).
Nutritional status and treatment on PD						
Umemoto et al. (17)	H&Y Disease duration	CONUT (Alb, TLC, T-cho), BMI, weight change, Swallowing	-	LEDD	One-way ANOVA, Bonferroni multiple comparisons, Spearman’s correlation.	93% of patients, were classified into normal nutrition and mild malnutrition groups by CONUT scores. The median weight loss of the DBS group was significantly lower than that of the oral medication alone group (*p* < 0.01). The weight loss had a significant correlation with disease duration (*p* = 0.04), the longer the duration of the disease, the greater the weight loss.
Batisse-Lignier et al. (18)	UPDRS	BMI, FFA, FFM, FM, EGP, GDRs, basal glucose and insulin plasma levels	–	–	ANOVA and “t” test.	EGP and GDR were higher in PD patients in OFF conditions than in the control group *p* < 0.05. Despite, no significant changes in blood glucose throughout the kinetic study, a significant and consistent 22% decrease in EGP occurred in PD patients during Stim-ON p < 0.01, and whole-body glucose kinetics in Stim-ON patients were no different from those of the control subjects.
Montaurier et al. (19)	UPDRS, H&Y, Schwab and England scale	BMI, FFM, Appendicular muscle mass, trunk fat-free and bone-free mas, FM, Trunk fat mass	–	LEDD, EE, SMR, BMR.	The “t” test, and the Pearson correlation.	Before surgery, male PD patients’ EE was higher while metabolic rate was lower compared to male controls, and BMR in “on” was higher than predictive basal metabolic rate but increased more without levodopa. EE during daily activities was greater “off” than “on” in both male and female groups. Following surgery, there was an increase in weight along with body fat and FFM in both men and women with Parkinson’s. SMR increased in men, but not in women. EE decreased significantly in both men and women, but there is no correlation between daily EE changes and weight gain.
Gut-brain axis on PD						
Heinzel et al. (20)	UPDRS Prodromal (ROMEIII, Sniffin Sticks) Medication UMSARS	BMI, Diet		Stool sample DNA extraction, Microbial measures (α and β diversity and abundance), enterotypes	Multifactorial analysis, linear regression, PERMANOVA, Fisher exact test, Kruskal-Wallis test, and multinomial logistic regression.	Diversity of microbiota α was related with physical inactivity (*p* = 0.007) and exposure to solvents. Diversity of microbiota β was related physical inactivity (*p* = 0.001), sex (*p* = 0.003), constipation (*p* = 0.002), rapid eye movement sleep disturbance (*p* = 0.037), and smoking (*p* = 0.020). Age and medication for lower urea were associated with *α*-*β* microbiota diversity. Constipation severity was significantly associated with decreased abundance of Faecalibacterium (*p* = 0.022) and Roseburia (*p* = 0.008), physical exhaustion with a decrease in Bifidibacterium (*p* = 0.039), and possible RBD with a decrease in Lactobacillus (*p* = 0.023). Subthreshold parkinsonism was associated with a decrease in Odoribacter (*p* = 0.031). Possible RBD was further associated with a decrease in Faecalicoccus (*p* = 0.017), and Victivallis (*p* = 0.017), and increased in the abundance of Haemophilus (*p* = 0.003). Urate-lowering medication was associated with a higher abundance of Closridium (*p* = 0.005) and Parasutterella (*p* = 0.032).
Jones et al. (21)	SCOPA-AUT UPDRS, H&Y		MoCA, HVLT, JOLO, SDMT, MCI	GI symptoms	Multilevel models	More severe GI symptoms are predictive of a less favorable trajectory on tests of written fluency, visuospatial, learning, and memory. Cognitive performance is only associated with GI symptoms and not related to autonomic non-gastrointestinal symptoms.

## 3 Results

The literature search retrieved 924 records, which were reduced to 846 after removing the duplicated ones (Figure 1). A meticulous title and abstract screening were done. After the title screening, 41 were included by title criteria inclusion, and title study exclusion criteria and excluded 600 such as those ones related to clinical trials or included participants with other diseases different to PD. Analyzing the abstracts, 205 manuscripts were excluded from the abstract; most of the studies were done with animals or were reviews. Finally, 14 fullpapers were eligible for our systematic review. Figure 1 shows the details of the screening process.

The sample size ranged from 16 to 666 patients, and the percentage of males oscillates between 39.2 to 70.8%. The mean age of PD patients ranged from 58.4 to 76.5 years old.

Table 1 includes the sociodemographic data and methodology of selected studies and Table 2 shows the outcomes and results of selected studies.

**Table 2:** Sociodemographic data and methodology of selected studies.

	Sample size			Age (Years)		Methods	
Study	Sample (% Males)	PD group	Control group	PD group	Control group	Objective	Type of study
Nutritional status and Qol							
Fernandez et al. (9)	23 (69.7%)	*N* = 33	–	58.9 ± 11.6	–	Evaluate the correlation between anthropometric variables and QoL of PD.	Cross-sectional, descriptive, and analytical study.
Sheard et al. (10)	74 (61.67%)	**Phase I**; Well-nourished *n* = 103 Moderately malnourished *n* = 17 **Phase** **II**; INT *n* = 10 SC *n* = 9	–	70 69.0	–	To determinate the relationship between nutritional status and QoL in PD.	Prospective study.
Nutritional status and motor and non-motor symptoms							
Nagano et al. (11)	53 (55.2%)	*N* = 61	–	76.5	-	Investigate the relationship between nutritional status and ADL in PD.	Retrospective cohort study.
Chia-yi Lien et al. (12)	15 (45.5%)	Risk of malnutrition *n* = 17 Well-nourished *n* = 16	–	Risk of malnutrition 71.65 ± 5.5 Well-nourished 71.50 ± 11.11	–	Evaluate the relationship between clinical symptoms severity and cognitive function of PD and serum vitamin D level and nutritional status.	Prospective study.
Fereshtehnejad et al. (13)	103 (68.7%)	Abnormal nutritional status *n* = 40 Normal nutritional status *n* = 10	–	Abnormal nutritional status 61.3 Normal nutritional status 61.3	–	To investigate the association of motor, psychiatric and fatigue features with nutritional status as well as the effects of malnutrition on different aspects of QoL in PD.	Prospective study.
Sheard et al. (14)	73 (58.4%)	Moderately malnourished *n* = 19 Well-nourished *n* = 106	–	Moderately malnourished 74.0 Well-nourished 69.0	–	Identify the determinants of nutritional status in PD.	Prospective study.
Nutritional status and PD severity							
Femat-Roldán et al. (1)	60 (51.72%)	*n* = 64	*n* = 52	67 ± 12	64 ± 12	Compare body composition and RMR between controls and PD.	Case–control study.
Pisciotta et al. (15)	124 (64%)	UPDRSIII<24 *n* = 97 UPDRSIII≥24 *n* = 98	–	UPDRSIII<2473.1 ± 6.7 UPDRSIII≥2474.2 ± 7.7	–	To investigate the association between PD severity and fat distribution patterns, and the potential modifier effect of nutritional status in this association.	Cross-sectional study.
Bril et al. (16)	60 (53%)	*N* = 114	–	66.1 ± 9.8	–	To analyze the relationship between weight loss, nutritional status, physical activity, and PD-related factors.	Prospective study.
Nutritional status and treatment on PD							
Umemoto et al. (17)	38 (39,02%)	DBS *n* = 34 LCIG *n* = 13 L-dopa *n* = 35	–	58.4 ± 10.2	–	Characterization of the impact of PD treatment on weight loss and clues to establish the administration of nutrition on PD.	Retrospective study.
Batisse-Lignier et al. (18)	8 (50%)	*n* = 8	*n* = 8	60.6 ± 2.7	64.6 ± 3.3	To analyze if the stimulation of the subthalamic nucleus affects post-absorptive glucose metabolism in PD.	Prospective study.
Montaurier et al. (19)	34 (70.8%)	*n* = 24	*n* = 24	61.1 ± 1.4	66.7 ± 0.9	Identify the mechanisms causing body weight gain in PD following DBS-STN.	Prospective study.
Gut-brain axis on PD							
Heinzel et al. (20)	350 (52.7%)	*N* = 666	–	68.4 ± 6.3	–	Evaluate the diversity, enterotype, and taxonomy of the intestinal microbiota and investigate its relationship with the risk of PD and its prodromes.	Prospective study.
Jones et al. (21)	277 (65.5%)	*N* = 423	–	6.2 ± 9.7	–	(1) Analyze the relationship between GI symptoms and neurocognitive tasks; (2) Analyze the relationship between GI symptoms and cognitive status.	Prospective study.

*AC, arm circumference; ACE-R, Addenbrooke’s cognitive examination; ADL, Activities of daily living; Alb, albumin; BCM, body cell mass; BDI, Beck’s Depression Inventory; BF, body fat percentage; BFM, body fat mass; BMC, bone mineral content; BMI, Body mass index; BMR, basal metabolic rate; CC, calf circumference; CFI, comparative fit index; CONUT, Controlling Nutritional Status; DBS, Deep brain stimulation; DBS-STN, Deep brain stimulation of the subthalamic nucleus; ECW, extracellular water; EE, energy expenditure; EGP, endogenous glucose production; ESR, erythrocyte sedimentation rate; EWB, emotional well-being; FFA, free fatty acid; FFM, Fat-free mass; FOG-Q, Freezing of gait Questionnaire; FSS, Fatigue Severity Scale; FIM, Functional Independence Measure; GDS, Geriatric Depression Scale; GDR, glucose disposal rate; GI, gastrointestinal; HADS, Hospital Anxiety and Depression Scale; h-s CRP, high-sensitive C-reactive protein; HVLT, Hopkins verbal learning test; H&Y, Hoehn and Yahr; INT, Intervention group; JOLO, judgment of line orientation; LCIG, Levodopa/carbidopa intestinal gel; L-Dopa, Levodopa; LEDD, levodopa equivalent daily dose; LM, lean mass; MCAS, Modified Constipation Assessment Scale; MCI, mild cognitive impairment; MDS-UPDRS, Movement disorder society unified Parkinson’s disease rating scale; MNA, Mini nutritional assessment; MMSE, Mini-Mental State Examination; MoCa, Montreal Cognitive Assessment; MSM, musculoskeletal mass; NFI, normed fit index; PD, Parkinson’s disease; PDQ-39, PD quality of life scale; PDSS-2, Parkinson’s disease sleep scale-2; PG-SGA, Patient-Generated Subjective Global Assessment; QUIP, questionnaire for impulsive-compulsive disorders in PD QoL, quality of life; REM, rapid eye movement; RMR, Resting metabolic rate; RMSEA, root mean square error of approximation; ROME III, Scale for diagnostic irritable bowel syndrome; SC, Standard care; SCOPA-AUT, Scale for outcomes in PD for autonomic symptoms; SDMT, symbol digit modalities test; SGA, Subject global assessment; SMR, sleep metabolic rate; STAI, Spielberg Trait Anxiety Inventory; T-cho, total cholesterol; TCS, triceps cutaneous skinfold; TLC, total lymphocyte count; UPDRS, Unified Parkinson’s disease rating scale; UMSARS, Unified multiple system atrophy rating scale; Vit, vitamin; VFA, visceral fat area; WC, waist circumference; WL, weight loss; YPAS, Yale physical assessment scale.

### 3.1 Relationship between nutritional status and QoL

Two studies examined the correlation between nutritional status and QoL in PD patients (9, 10) Fernandez et al. (9) investigated anthropometric variables’ relationship with PD patients’ QoL. They found a lower perception of QoL in the dimension of body discomfort (75.3 +/− 16.6), social support (62.7 +/− 15.7), and mobility (61.0 +/− 23.6), and that older age correlated with lower scores in mobility (*p* = 0.005), daily physical activity (*p* = 0.016), communication (*p* = 0.030), body discomfort (*p* = 0.008), and, overall, PD QoL scale (PDQ-39) score (*p* = 0.024). Additionally, body mass index (BMI) correlated with social support (*p* = 0.000) and cognition (*p* = 0.025) dimensions, while triceps cutaneous skinfold (TCS) correlated with daily physical activity (*p* = 0.029), cognition (*p* = 0.016), and social support (*p* = 0.013). Age remained a significant predictor of QoL scores even after accounting for gender (*p* = 0.043) and BMI (*p* = 0.047), suggesting age-related effects independent of nutritional status.

Secondly, Sheard et al. (10) evaluated the relationship between nutritional status and QoL and the effect of a nutrition intervention on QoL in PD. In phase I of the study, there was a significant correlation between Patient-Generated Subjective Global (PG-SGA) and PDQ-39 scores (*p* = 0.002), indicating that poorer nutritional status was associated with poorer QoL. The QoL was poorer in the malnourished group, particularly for mobility (*p* = 0.016) and activities of daily living (ADL) (*p* = 0.008). In phase II, they analyzed 2 groups, [intervention group (INT) and standard care group (SC)] but no significant differences were observed between groups in QoL (PDQ-39 total or sub-scores). There was a positive relationship between changes in PG-SGA and PDQ-39 scores during the 12-week intervention period, regardless of the group (SC or INT). The INT group demonstrated a trend toward greater improvement than the SC group in the majority of domains, which resulted in clinically significant changes. QoL scores improved in both groups over the 12 weeks. However, the changes were not statistically significant. In summary, malnourished people with PD had a poorer QoL than well-nourished, this was particularly evident in the areas of mobility and ADL. The was a borderline significant relationship between improvements in nutritional status during the nutrition intervention and improvements in QoL. Furthermore, the nutrition intervention resulted in improvements in emotional well-being.

### 3.2 Relationship between nutritional status and motor and non-motor symptoms

Four studies observed the relationship between nutritional status and motor and non-motor symptoms (11–14). Firstly, Nagano et al. (11) focused on nutritional status and ADL in PD patients. They evaluated nutritional status with Controlling Nutritional Status (CONUT) method, its scores albumin (Alb), total lymphocyte count (TLC), and total cholesterol (T-cho) values, and to evaluate the ADL they used the motor subdomains of the Functional Independence Measure (FIM). Variables that were significantly associated with the FIM gain, included PD severity (*p* < 0.001), dysphagia (*p* < 0.005), cognitive FIM (*p* < 0.089), and grip strength (*p* < 0.080). A poor nutritional status (CONUT score > 3) was significantly associated with poor FIM gain. A CONUT score of 3 was the change point from which the ADL of patients with PD decreased significantly as the scores increased. Poor nutritional status (CONUT score > 3) was significantly associated with poor ADL in PD patients.

Secondly, Chia-Yi Lien et al. (12) evaluated the relationship between the severity of clinical symptoms and cognitive function of PD patients, the serum vitamin D level, and nutrition status. They enrolled 33 patients and after the initial nutritional assessment, they divided them in well-nourished status and risk of malnutrition status. The group at risk of malnutrition had a higher Unified Parkinson’s Disease Rating Scale (UPRDS) score (*p* < 0.046), and a significant impairment in memory (*p* < 0.002), and calculation (*p* < 0.010). Patients with lower serum vitamin levels had a higher high-sensitive C-reactive protein (hs-CRP) level which influenced the cognitive function of PD patients. Therefore, abnormal serum vitamin D levels may have an indirect influence on the cognitive function of PD patients through the influence on the hs-CRP level.

Thirdly, Fereshtehnejad et al. (13) investigated the relationship between motor, psychiatric, and fatigue features with nutritional status, as well as the effects of malnutrition on different aspects of QoL in PD. Out of 150 PD, 37 patients were at risk of malnutrition and 3 were malnourished. The lower nutritional status [Mini Nutritional Assessment (MNA)], the higher the total score in the UPDRS (*p* < 0.001) and PD duration (*p* = 0.002). Patients with abnormal nutritional status had a significantly longer history of PD compared to those with normal nutritional status (*p* = 0.045). Additionally, among patients with abnormal nutritional status, the median score of Hoehn and Yahr (H&Y) stage was significantly higher (*p* < 0.001). Moreover, severe anxiety (*p* = 0.002), depression (*p* < 0.001), and fatigue (*p* < 0.001) were observed more frequently in this group. In addition, the weight-adjusted levodopa dose was inversely correlated with total MNA. This total MNA mean was significantly lower among the female PD patients compared to males (*p* = 0.002). Higher scores (worse conditions) of PDQ-39 were observed in emotional well-being (*p* < 0.001) and mobility domains (*p* < 0.001). Except in the stigma domain, all the PDQ-39 domains showed that higher scores (indicating worse QoL) were associated with lower scores on the MNA scale (indicating poorer nutritional status).

Fourthly, Sheard et al. (14) identified which factors predicted nutritional status in patients with PD free-living in the community when measured by the Subjective Global Assessment (SGA). The 15% of PD patients were moderately malnourished. They divided the patients into two groups study (well-nourished and moderately malnourished patients). The majority of the malnourished were adults (range 35–92 years) (81%), and had more severe disease, while the moderately malnourished group had higher UPDRS II (*p* = 0.009) and UPDRS III (*p* = 0.008) scores, levodopa equivalent daily dose (LEDD)/body weight (*p* = 0.022). Regarding cognition, the malnourished patients’ scores were significantly lower in the Mini-Mental State Examination (MMSE) (*p* = 0.009), visuospatial (*p* = 0.013), and attention and orientation (*p* = 0.002), but not on Addenbrooke’s Cognitive Examination (ACE) total score. There were significantly higher depression scores [Beck’s Depression Inventory (BDI)] (*p* = 0.001) and the sub-score in gastrointestinal symptoms of Scales for Outcomes in Parkinson’s disease-Autonomic (SCOPA-AUT) (*p* = 0.046). The malnourished group scored poorer on the majority of the assessments than did well-nourished. More severe motor symptoms and more depressive symptoms were predictive of malnutrition and a higher PG-SGA score. Other factors that contributed, were older age of diagnosis, higher LEDD/weight, and living alone.

### 3.3 Relationship between nutritional status and PD severity

Three studies analyzed the relationship between nutritional status and PD severity (weight-related mobility impairments) (1, 15, 16). Firstly, Femat-Roldán et al. (1) compared body composition and resting metabolic rates (RMR) between PD patients and healthy controls. They analyzed two subgroups within the PD patients: tremor dominant (PD-TD) and postural instability/gait difficulty (PD-PIGD). PD patients exhibited lower body fat mass, body fat percentage, visceral fat area, waist circumference, and arm circumference compared to controls, with no significant differences in certain UPDRS scores (I and IV) but notably higher scores in parts II and III for the PD-PIGD group. PD-PIGD patients showed lower body fat mass (*p* = 0.020), body fat percentage (*p* = 0.001), waist circumference (*p* = 0.014), and visceral fat area (*p* = 0.029) compared to PD-TD patients, along with higher fat-free mass (*p* = 0.049). RMR (*p* = 0.010) was higher in PD patients compared to controls, particularly in the PD-PIGD group (*p* = 0.001), potentially leading to a selective reduction in body fat mass (*p* = 0.001) without affecting musculoskeletal mass. Weight loss in PD patients appears to be a complex outcome involving multiple factors.

Secondly, Pisciotta et al. (15) investigated the PD severity and several parameters of adiposity, paying special attention to the topology of fat distribution (android versus gynoid), and to address the potential modifier effect of nutritional status in the association between PD severity and fat distribution. They did two groups and compared participants with a UPDRS III below and above the median value (UPRDS III = 24). The group who had a UPDRS <24 presented better cognitive function, mood, functional status, nutritional status, lower erythrocyte sedimentation rate (ESR) values, and comorbidities, higher trunk-leg and trunk-limb ratios. A higher score on the UPDRS III was associated with lower total body fat, android fat, trunk-leg fat ratio, trunk-limb fat ratio, and android-gynoid ratio. They showed a significant correlation between all the adiposity parameters and UPDRS III scored stratified by the MNA.

Thirdly, Bril et al. (16) studied the relationship between body weight, nutritional status, physical activity, and PD-related factors, finding that longer disease duration was negatively related to nutritional status (*p* = 0.01), and nutritional status, had positively related to body weight (*p* = 0.01). PD severity (UPDRS II and III score) was associated with reduced cognitive function (*p* = 0.01) which was a positively related to nutritional status (*p = 0.*01). In addition, nutritional status was related to body weight (*p* < 0.01). Binge eating (*p* = 0.001) as much as physical activity (*p* = 0.001) were also directly, and positively related to body weight in their sample of patients with PD. Nutritional status, binge eating, and physical activity were the only variables associated directly with body weight. Disease duration, UPDRS II and III scores, and cognitive function were indirectly associated with body weight, but through indirect influence on nutritional status.

### 3.4 Relationship between nutritional status and PD treatment

One study analyzed the impact of each treatment [Deep Brain Stimulation (DBS), levodopa/carbidopa intestinal gel (LCIG), and oral medication (L-dopa)] on weight loss, and two studies were related to the stimulation of the Subthalamic Nucleus-Deep Brain Stimulation (STN-DBS) with changes in the metabolism of glucose and energy expenditure (EE) (17–19). Firstly, Umemoto et al. (17) compared oral medication and device-assisted therapies in PD like DBS, and LCIG. Most of the patients (93%) were classified into normal nutrition and mild malnutrition categories according to the CONUT score. Only 6 patients (7%) were classified as moderately or severely malnourished. The DBS group showed a lower median in H&Y (*p* < 0.01), age onset (*p* < 0.01), and videofluoroscopic dysphagia scale (VDS) (*p* < 0.05), and the LCIG group had a higher median in LEDD in comparison to the other groups (*p* < 0.01). The median of weight loss per year was 0.23 kg in the DBS group, 0.82 kg in the LCIG group, and 1.27 kg in the oral medication alone group. They found a significant correlation between the rate of weight loss and follow-up period (*p* < 0.022) in the patients who received oral treatment alone. On the other hand, DBS patients showed weight gain within 5 years after surgery, and after that, they decreased body weight gradually.

Secondly, Batisse-Lignier et al.[18]hypothesized that STN-DBS might affect postabsorptive glucose metabolism in PD. Endogenous glucose production (EGP), and glucose disposal rates (GDR) were higher in PD patients in Stim-off conditions than in the control group (2.62+/−0.09 vs. 2.27+/− 0.10 mg/kg. min*, p* < 0.05). There were no significant changes in blood glucose during the kinetic study and a significant and consistent 22% decrease in EGP in patients with PD in Stim-on (2.04+/−0.07 mg/kgˉ^1^.minˉ^1^; *p* < 0.01). They found that DBS in PD patients affect EGP, glucose disposal, suggesting that a cross talk between the central and peripheral tissues may regulate glucose homeostasis.

Thirdly, Montaurier et al. (19).They studied PD patients before and after surgery. They included 17 men and 7 women. The month before surgery, in men (but not in women) with PD, the daily EE was higher, while the sleep metabolic rate (SMR) was lower compared to matched healthy men. (+ 9.2+/−3.9 and-8.2 +/− 2.3%, respectively, *p* < 0.05), and basal metabolic rate (L-dopa “on”) was higher than predicted basal metabolic rate (+11.5+/− 4.0%, *p* < 0.05), but was further increased without L-dopa (+8.4+/−3.2% vs. L-dopa “on” *p* < 0.05). EE during daily activities was higher during “OFF” periods compared to “ON” for both men (+19.3+/−3.3%, *p* < 0.001) and women (+16.1+/−4.7%, *p* < 0.01). After 3 months from the surgery, there was a 3.4 +/−0.6 kg (*p* < 0.001) body weight increase together with fat mass (*p* < 0.001) and fat-free mass (*p* < 0.05) in women with PD. SMR increased in men (+7.5 +/− 2.0%, *p* < 0.01) to reach control values but no changes in women. Daily EE was significantly reduced in men and women (−7.3+/− 2.2% and-13+/− 1.7%, respectively, *p* < 0.01). PD was related with alterations in energy metabolism that were normalized after DBS-STN surgery while energy intake was maintained. There were significant inter-individual variations and gender-related differences in the quality of body weight gain: men mainly gained a free mass of fat and women only in fat. Besides, progressive physical training in the early days following surgery may help limit weight gain in some patients.

### 3.5 Gut-brain axis in PD

Two other studies analyzed the changes in the gut health and microbiota, and their relationship with PD. (20, 21) Firstly, Heinzel et al. (20) investigated intestinal microbial diversity, enterotypes, and taxonomic composition in relation to risk and prodromal markers of PD, as well as overall prodromal risk and probability. They analyzed a sample of elderly individuals and found that physical inactivity, occupational solvent exposure, certain medications such as those for thyroid and uric acid reduction, as well as exhaustion from climbing stairs, were inversely associated with alpha microbial diversity (*α*-diversity). Conversely, the severity of constipation (*p* < 0.045) and age (*p* < 0.047) showed positive associations with α-diversity. Beta microbial diversity (*β*-diversity) showed significant associations with various risk factors and prodromal markers of PD, including age, physical inactivity, constipation, BMI, sex, smoking, rapid eye movement sleep behavior disorder (RBD), different medications, and consumption of dark bread. Although motor deficits had no effect on their own, the interaction between physical inactivity and motor deficits explained some of the variability in β-diversity (*p* = 0.002). Enterotypes also exhibited differences in terms of risks and prodromal markers: lower levels of physical inactivity and more severe constipation were observed in the Firmicutes-enriched enterotype, while higher levels of physical inactivity were associated with the Bacteroides-enriched enterotype. Additionally, certain microbial taxa were associated with specific variables, such as a decreased abundance of Faecalibacterium (*p* = 0.022) and Roseburia (*p* = 0.008). Furthermore, physical exhaustion with a decrease in Bifidobacterium (*p* = 0.039), and possible RBD with a decrease in Lactobacillus (*p* = 0.023), Faecalicoccus (*p* = 0.017), and Victivallis (*p* = 0.017), and with an increase in abundance of Haemophilus (*p* = 0.039). Motor deficits were associated with a decrease in Odoribacter (*p* = 0.031). Urate-lowering medication was associated with a higher abundance of Clostridium III (*p* = 0.005) and Parasutterella (*p* = 0.032). They found that several risk and prodromal markers of PD were associated with gut microbiome composition, in particular, markers related to motor aspect and constipation, were associated with altered microbial *α* and *β*-diversity, enterotypes and bacterial abundance. Constipation, physical inactivity, possible RBD, urate levels, smoking, and subthreshold parkinsonism might be particularly linked to the prodromal microbiome in PD.

Secondly, Jones et al. (21) investigated how the severity of gastrointestinal (GI) symptoms relates to cognitive impairment in newly diagnosed PD patients. They found that patients with more severe GI symptoms exhibited a more pronounced cognitive decline. GI symptoms had a significant impact on various cognitive functions: global cognitive functions (*p* = 0.018), working memory (*p* = 0.033), processing speed (*p* = 0.034), verbal comprehension (*p* = 0.016), and delayed verbal recall (*p* = 0.002). Additionally, they observed that more frequent GI symptoms were associated with a higher risk of mild cognitive impairment (MCI) and PD dementia (PDD), with these effects being particularly pronounced in men (*p* = 0.001), older individuals (*p* < 0.001), and those with a lower educational level (*p* = 0.001). There was a significant relationship between gut health and cognitive functioning through the gut-brain axis among PD patients. More frequent gastrointestinal symptoms were predictive of worse performance across all cognitive domains and were risk factors for PD-MCI or PDD. The presence of gastrointestinal symptoms may serve as an early marker of cognitive impairment in PD.

## 4 Discussion

Therefore, this review aims to evaluate whether nutritional alterations are related either to the severity of motor and non-motor symptoms through the gut-brain axis or to the different treatments for PD and whether all of this, in turn, impacts the QoL of patients. In this review, we observed that alterations in nutritional status influenced both motor and non-motor symptoms (psychiatric disturbances and fatigue), thus affecting the severity of PD symptoms and impacting QoL. Alterations in the QoL of PD patients particularly affect dimensions such as discomfort, communication, ADL, and mobility (9, 13, 20)

Patients with PD tended to be underweight or malnourished, which may be associated with increased EE caused by the progression of motor symptoms, as well as GI symptoms and anorexia derived from the side effects of PD treatments. Thus, altering the patient’s nutritional status (22). All these factors and their impact on nutritional status may in turn interfere with the QoL of PD patients. According to Sheard et al. (10) and Fernández et al. (9), nutritional status is significantly related to quality of life (QoL). Malnourished patients had lower QoL in most domains of the PDQ-39, particularly noticeable in areas such as mobility, bodily discomfort, daily physical activities, social support, and communication. Overall, the studies highlighted the complexity of the relationship between nutritional status and various aspects of QoL in PD patients, emphasizing the need to consider nutritional factors in their care and management (13, 21).

Regarding nutritional status and its impact on motor and non-motor symptoms such as psychiatric symptoms, cognitive impairment, and fatigue; many motor, psychiatric, and fatigue symptoms were significantly associated with nutritional status in patients with PD. As described by Golman et al. (5), there was a high incidence of mood disorders (depression, anxiety, apathy) in this pathology, and up to 85% of those affected had hyposmia or anosmia from the early stages of the disease, affecting appetite. Non-motor symptoms such as psychiatric symptoms (depression and anxiety) were associated with nutritional status in these patients, and observed, how different aspects of health were related to QoL, especially emotional well-being and mobility (13). While focused on cognition, could be indirectly influenced (by elevated levels of hs-CRP) due to abnormal serum levels of vitamin D in PD. These patients experienced nutritional status alteration that would both significantly deteriorate cognition in the domains of memory and calculation and worsen motor symptoms (12). However, according to Brill et al. (16), the only variables that were directly associated to QoL were nutritional status, binge eating, and physical inactivity, while disease duration, UPDRS II and III scores, and cognitive impairment also influenced body weight, but through an indirect influence on nutritional status.

Focusing on severity, studies found that more severe symptoms, lower total fat mass, and depressive symptoms predicted malnutrition (1, 13–16). As Femat-Roldán et al. (1), demonstrated, PD patients with lower body fat mass had higher scores on the UPDRS II and III scales, thus making weight loss and malnutrition two of the most frequently observed non-motor symptoms in PD and associated with poorer QoL. Similarly, Pisciotta et al. (15) also concluded that good nutritional status could protect PD patients from weight loss related to the severity of motor symptoms. Taking it a step further, Brill et al. (16), Fereshthnejad et al. (13), and Sheard et al. (14) correlated nutritional status with symptom severity as well as cognitive status. They observed that motor and psychiatric symptoms (such as depression, anxiety, and fatigue) were associated with the nutritional status of PD patients, thereby impacting QoL.

Regarding weight fluctuations, these could have therapeutic and prognostic implications: lower weight was associated with a higher incidence of motor complications of dopaminergic treatment (dyskinesias) and the risk of overall deterioration with complications (3). In the same line, weight loss and malnutrition had a negative impact on PD QoL, and Sepulveda-Contardo et al. (7) pointed out that this was more common in the advanced stages of the disease. PD treatments were also related to nutrition in QoL. Unemoto et al. (17), pointed out that patients under oral medications were who had a higher weight loss per year, followed by LCIG and DBS. In fact, the DBS group showed weight gain within 5 years after surgery and then gradually decreased body weight. This could be due to initial control of EE and subsequently adjusting the frequency of DBS. Regarding the LCIG group, weight loss could be due to the constant infusion of medication, which could interfere with the absorption process of different nutrients, which in turn could influence weight. Another factor that may influence weight loss is the control of dyskinesias in this group. Patients on oral medication may lose more weight as levodopa in the early stages may be poorly tolerated and cause nausea and vomiting.

Several studies, including Batisse-Lignier et al. (18), noted that DBS affects glucose metabolism control by regulating EGP independently of plasma glucose levels and pancreatic hormones in PD patients. EGP was observed to be higher when stimulation was turned off and normalized to levels similar to control groups when turned on, suggesting a link between central nervous system activity and peripheral tissues involved in glucose homeostasis. Furthermore, as noted by Umemoto et al. (17) and Montaurier et al. (19), post-surgical normalization of energy metabolism can lead to weight gain, with differing impacts based on gender; men tend to gain lean body mass, whereas women primarily gain fat. Various factors contribute to weight gain post-DBS, including improved swallowing, enhanced ability to handle food due to reduced movement issues, less nausea and anorexia linked to dopaminergic treatments, increased appetite, and altered metabolic control due to specific surgical target effects. Additionally, changes in physical activity can help mitigate weight gain, contributing significantly to patients’ QoL improvements when managed effectively.

Given the existing relationship in the gut-brain axis, it is observed that gastrointestinal alterations are due to intestinal dysbiosis, where there is a disturbance of the microbiota (in its diversity, enterotypes, and taxonomic composition) that may lead to neurological compromises with subsequent cognitive impairment and could be analyzed using prodromal markers of this disease and other neurodegenerative disorders. These microbiota alterations could be caused by both the type of nutrition and the type of treatment these patients receive. Prolonged alteration of nutritional status and the microbiota could influence both motor and non-motor symptoms of the disease, and therefore the QoL in PD, either by producing intestinal alterations, affecting activity and physical condition, as well as cognitive status and perception of QoL. In this regard, Jones et al. (21), observed that GI alterations produced dysbiosis of the intestinal microbiota, which could subsequently lead to increased neuroinflammation and degeneration of the neural system important for cognitive functioning. Regarding microbiota diversity, Heinzel et al. (20), observed several risk and prodromal markers (particularly related to motor aspects and constipation) in PD that were associated with altered *α* and *β* diversity of the microbiota, enterotypes, and bacterial abundance. Therefore, physical inactivity, constipation, possible RBD, smoking, and subthreshold Parkinsonism had an impact on alterations in the microbial community composition with different microbial measures.

The reviewed studies identified several limitations that should also be taken into account. One limitation was the small sample size of most of the studies. Additionally, various types and stages of PD were examined across the studies which can be challenging when trying to compare results. Secondly, PD is a slowly progressive neurodegenerative disease, and studies that last longer would be needed to better observe the impact of nutritional status in the different stages of the disease. Thirdly, apart from L-dopa treatment, there was a lack of data on the usual treatment of PD patients. Fourthly, some studies did not consider factors that may influence weight gain, such as diet type, dysautonomic symptoms, hormonal factors, or lack of physical activity.

Considering that there are some prodromal symptoms in PD at the intestinal level, such as alteration in the composition of the intestinal microbiota and deposition of alpha-synuclein, at some point before the disease develops, and that the degree of nutritional status may influence the severity of motor symptoms of the disease, additional studies should be conducted on fecal microbiota to better understand how to improve the gut-brain relationship, as well as nutritional studies and how nutrition in these patients can modify the microbiota, thus acting on their QoL. Another factor to consider is how the treatments may modify the nutritional status of patients or alter their microbiota. Future studies should aim to achieve early stages for PD patients.

## 5 Conclusion

In conclusion, given the relationship between nutritional status and the QoL in PD patients and the existing relationship in the gut-brain axis, better nutritional status would maintain a balanced intestinal microbiota and delay cognitive decline, thus helping to improve ADL and ultimately enhancing the QoL of PD patients. This systematic review confirmed that the nutritional status of PD patients influences both motor and non-motor symptoms of the disease, as well as intestinal microbiota and ADL, and therefore QoL.

Understanding the contribution of optimal nutritional status and intestinal health to cognitive decline is important, as it will help to make a good prognosis and intervention (fecal transplant, administration of prebiotics/probiotics, personalized diets, adjustments in treatments received). Therefore, it is essential to follow a balanced diet adapted and personalized to the patient’s needs, as not everyone has the same EE, the same difficulty in eating, and there is a wide variety of microbial diversity. For this, it would be important to have a dietitian on the medical team, to be able to identify possible nutritional alterations early and to intervene on them in a multidisciplinary way (23).

## Data Availability

The raw data supporting the conclusions of this article will be made available by the authors, without undue reservation.
