# Supplementary material for: Implication of nutrition in severity of symptoms and treatments in quality of life in Parkinson’s disease: a systematic review
Source: Front Nutr. 2024 Oct 8;11:1434290. doi: 10.3389/fnut.2024.1434290 (PMC11493749; doi:10.3389/fnut.2024.1434290)
Supplement: Supplementary file 1 [file Data_Sheet_1.zip › Abbreviations.DOCX]

**Supplementary material 1.**

**Notes: ***Abbreviations:

AC: arm circumference.

ACE-R: Addenbrooke`s cognitive examination.

ADL: Activities of daily living.

Alb: albumin.

BCM: body cell mass.

BDI: Beck`s Depression Inventory.

BF: body fat percentage.

BFM: body fat mass.

BMC: bone mineral content.

BMI: Body mass index.

BMR: basal metabolic rate.

CC: calf circumference.

CFI: comparative fit index.

CONUT: Controlling Nutritional Status.

DBS: Deep brain stimulation.

DBS-STN: Deep brain stimulation of the subthalamic nucleus.

DXA: dual-energy X-ray absorptiometry.

ECW: extracellular water.

EE: energy expenditure.

EGP: endogenous glucose production.

ESR: erythrocyte sedimentation rate.

EWB: emotional well-being.

FFA: free fatty acid.

FFM: Fat-free mass.

FOG-Q: Freezing of gait Questionnaire.

FSS: Fatigue Severity Scale.

FIM: Functional Independence Measure.

GDS: Geriatric Depression Scale.

GDR: glucose disposal rate. GI: gastrointestinal.

HADS: Hospital Anxiety and Depression Scale.

h-s CRP: high-sensitive C-reactive protein.

HVLT: Hopkins verbal learning test.

H&Y: Hoehn and Yahr.

INT: Intervention group.

JOLO: judgment of line orientation.

LCIG: Levodopa/carbidopa intestinal gel.

L-Dopa: Levodopa.

LEDD: levodopa equivalent daily dose.

LM: lean mass.

MCAS: Modified Constipation Assessment Scale.

MCI: mild cognitive impairment.

MDS-UPDRS: Movement Disorder Society Unified Parkinson’s disease rating scale.

MNA: Mini nutritional assessment.

MMSE: Mini-Mental State Examination.

MoCa: Montreal Cognitive Assessment.

MSM: musculoskeletal mass.

NFI: normed fit index.

PD: Parkinson’s disease.

PD-PIGD: PD postural instability/gait difficulty.

PDQ-39: PD quality of life scale.

PDSS-2: Parkinson´s disease sleep scale-2.

PD-TD: PD tremor dominant.

PG-SGA: Patient-Generated Subjective Global Assessment.

QUIP: questionnaire for impulsive-compulsive disorders in PD.

QoL: quality of life.

RBD: rapid eye movement sleep behavior disorder.

REM: rapid eye movement.

RMR: Resting metabolic rate.

RMSEA: root mean square error of approximation.

ROME III: Scale for diagnostic irritable bowel syndrome.

SC: Standard care.

SCOPA-AUT: Scale for outcomes in PD for autonomic symptoms.

SDMT: symbol digit modalities test.

SGA: Subject global assessment.

SMR: sleep metabolic rate.

STAI: Spielberg Trait Anxiety Inventory.

T-cho: total cholesterol.

TCS: triceps cutaneous skinfold.

TLC: total lymphocyte count.

UPDRS: Unified Parkinson’s disease rating scale.

UMSARS: Unified multiple system atrophy rating scale.

Vit: vitamin.

VFA: visceral fat area.

WC: waist circumference.

WL: weight loss.

YPAS: Yale physical assessment scale.
