# Supplementary material for: Implication of nutrition in severity of symptoms and treatments in quality of life in Parkinson’s disease: a systematic review
Source: Front Nutr. 2024 Oct 8;11:1434290. doi: 10.3389/fnut.2024.1434290 (PMC11493749; doi:10.3389/fnut.2024.1434290)
Supplement: Supplementary file 1 [file Data_Sheet_1.zip › Keyword strategy.DOCX]

Supplementary material 3. Keywords strategy.

|  | **Database** | |  |
| --- | --- | --- | --- |
| **Keywords** | ***MEDLINE*** | ***EMBASE*** | ***MENDELEY*** |
| (Parkinson’s disease) AND (quality of life) AND (nutritional alterations) OR (nutritional status) OR (nutrition disorders) OR (nutritive value) AND  (Deep brain stimulation) AND  (Dysphagia) | 10 | 0 | 11 |
| (Parkinson’s disease) AND (quality of life) AND (nutritional alterations) OR (nutritional status) OR (nutrition disorders) OR (nutritive value) | 13 | 55 |  |
| (Parkinson’s disease) AND (nutritional alterations) OR (nutritional status) OR (nutrition disorders) OR (nutritive value) | 499 | 336 |  |
